# Recipient of 2019 CSSA Editor's Citation for Excellence named

**DOI:** 10.1002/tpg2.20024

**Published:** 2020-05-12

*The editorial board of The Plant Genome is pleased to announce the recipient of the 2019 Editor's Citation for Excellence. These awards recognize the outstanding professional commitment and dedication of volunteer reviewers and/or editors who, through their excellent insights and comments, have helped maintain the high standard and quality of papers published in the journal. Recipients were nominated based on their thorough, competent, and timely reviews or editing of manuscripts. Each will receive a certificate of appreciation, an ASA‐CSSA‐SSSA gift certificate, and recognition in CSA News*.

**Figure:**
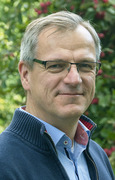
Nils Stein, Leibniz Institute of Plant Genetics and Crop Plant Research, Gatersleben

